# Treatment patterns and use of healthcare resources of patients with atherosclerotic cardiovascular disease and hypercholesterolemia and patients with familial hypercholesterolemia in Spain: Protocol of the Reality study

**DOI:** 10.3389/fcvm.2022.966049

**Published:** 2022-08-04

**Authors:** Vivencio Barrios, Mar Castellanos, Raquel Campuzano Ruiz, Jorge Francisco Gómez Cerezo, Isabel Egocheaga Cabello, José M. Gámez, Icíar Martínez López, José María Mostaza, Nuria Morant Talamante, Javier Parrondo, Aram Sicras Navarro, Inés Pérez Román, Antoni Sicras-Mainar, Vicente Pallarés-Carratalá

**Affiliations:** ^1^Cardiology Service, University Hospital Ramón y Cajal, Madrid, Spain; ^2^Department of Medicine and Medical Specialties, University of Alcalá de Henares, Madrid, Spain; ^3^Neurology Service, Complejo Hospitalario Universitario A Coruña, A Coruña, Spain; ^4^Cerebrovascular Diseases: Clinical and Translational Neurology, Instituto de Investigación Biomédica de A Coruña, A Coruña, Spain; ^5^Cardiac Rehabilitation, Cardiology Service, Hospital Universitario Alcorcón, Madrid, Spain; ^6^Internal Medicine Service, Hospital Universitario Infanta Sofía, Madrid, Spain; ^7^Family Medicine Service, Centro de Salud Isla de Oza, Madrid, Spain; ^8^Cardiology Service, Hospital Universitario Son Llátzer, Palma, Spain; ^9^Department of Medicine, Universidad de las Islas Baleares, Palma, Spain; ^10^Pharmacy Service and Molecular Diagnostics and Clinical Genetics Unit, Hospital Universitario Son Espases, Palma, Spain; ^11^Internal Medicine Section, Hospital Universitario La Paz, Madrid, Spain; ^12^Medical Department, Novartis Pharmaceuticals, Barcelona, Spain; ^13^Health Economics Department, Novartis Pharmaceuticals, Barcelona, Spain; ^14^Health Economics and Outcomes Research Department, Atrys Health, Barcelona, Spain; ^15^Health Economics and Outcomes Research Department, Atrys Health, Madrid, Spain; ^16^Unidad de Vigilancia de la Salud, Unión de Mutuas, Castellón, Spain; ^17^Departamento de Medicina, Universitat Jaume I, Castellón, Spain

**Keywords:** hypercholesterolemia, atherosclerosis, hypolipidemic agents, health resources, health care costs

## Abstract

**Background:**

Atherosclerotic cardiovascular diseases (ASCVD) and dyslipidemia are associated to a higher risk of cardiovascular events, mortality, use of healthcare resources and costs. In Spain, the evidence about the administration of lipid-lowering treatments in clinical practice, and their clinical effectiveness in patients with ASCVD and hypercholesterolemia and patients with FH is scarce. Therefore, a multidisciplinary working group of cardiologists, family physicians, internal medicine specialists and neurologists was gathered for the Reality study. The aim of this study is to describe the demographic and clinical characteristics, comorbidities, and concomitant medication of patients with ASCVD and hypercholesterolemia and of patients with familial hypercholesterolemia (FH). The use of healthcare resources and costs associated to the management of these diseases after their diagnosis were also considered.

**Methods:**

This is an observational and retrospective study, based on the BIG-PAC^®^ database, which includes the electronic medical registries (EMRs) of 1.8 million people from 7 Autonomous Communities in Spain (including public primary care centers and hospitals). The study includes patients who had a new or recurrent episode of ASCVD during the recruitment period (from 01/01/2017 to 31/12/2018). The index date will be defined as the date of the ASCVD event, and the follow-up period will be 24 months. According to their first diagnosis in the database, patients will be classified as ASCVD (5 groups: stable/unstable angina, acute myocardial infarction, ischemic stroke, transient ischemic attack, and peripheral arterial disease) or FH.

**Discussion:**

This study aims to analyze the treatment patterns and use of healthcare resources of ASCVD and FH in Spain. The prevalence of these disorders will also be estimated. Due to the high morbidity and mortality associated with these diseases, it is expected that our study will provide useful information for healthcare systems and decision makers to improve the management of these disabling diseases.

## Introduction

Cardiovascular (CV) diseases are due to disorders of the heart and blood vessels, such as myocardiopathy, ischemic cardiopathy, cerebrovascular accident and peripheral artery disease (1, 2). Atherosclerosis is the most frequent cause of vascular disease (3), and is usually related to lifestyle and biochemical and physiological modifiable factors like excess weight or high blood pressure or high lipid levels (4). The reduction of CV risk may decrease the morbidity and mortality associated with ASCVD (1, 3), which constitutes one of the main causes of morbimortality and premature deaths in developed countries (1, 2), leading to a high use of resources and costs for healthcare systems (3–5).

Dyslipidemia is caused by abnormal levels of lipoproteins in blood, which is one of the main risk factors of coronary disease in adults (3, 6, 7). The prevention and control of these diseases is critical for healthcare systems (1, 6, 7). The aim of secondary prevention is to stabilize the atherosclerotic plaque, by reducing the inflammation, the risk of rupture and the subsequent thrombosis (1, 3). The pharmacological treatment is one of the main components of the therapy; lipid-lowering drugs, particularly statins (HMG-CoA reductase inhibitors), have shown to reduce CV risks (1, 2, 7, 8). In addition, several studies showed that in patients who received treatments for secondary prevention, the lower the blood levels of lipoproteins of low density (LDL) are, the better prognosis patients have (9). In this sense, a meta-analysis showed that major CV events (major coronary events, coronary revascularization, or stroke) were reduced by 22% (non-fatal ischemic stroke, 23%; deaths due to coronary disease, 20%; deaths due to other cardiac causes, 11%) for 5 years per each 1 mmol/L reduction in LDL (3, 10).

Despite these results, the percentage of patients who meet the definition of LDL control is still low (3, 11–14). The EUROASPIRE study (14) showed that in patients who had suffered a coronary event, 71% of them had LDL levels ≥70 mg/dL, while 37% of them had LDL levels ≥100 mg/dL. In addition, the Da Vinci study confirmed that only 18% of patients on secondary prevention treatments reach LDL levels <1.4 mmol/L (55 mg/dL) (15). In this sense, some groups of patients may have difficulties to achieve the therapeutic goals, such as those diagnosed with familial hypercholesterolemia (FH), those with partial or total intolerance to statins and/or very-high CV risk. Although statins are safe and well tolerated, their use at high doses and/or their long-term administration may increase the risk of adverse events, like elevated transaminase levels or myopathies. The lack of LDL control is associated with the inadequacy of treatment, the lack of efficacy of lipid-lowering drugs combinations and the low adherence to medication (11, 13, 16, 17).

In Spain, the evidence available about the administration of lipid-lowering treatments in clinical practice, and their clinical effectiveness in patients with ASCVD and hypercholesterolemia and patients with FH is scarce. However, this information is likely to be of particular interest for healthcare systems, along with the analysis of the use of healthcare resources and the treatment and follow-up costs of these patients. Therefore, a multidisciplinary working group of cardiologists, family physicians, internal medicine specialists and neurologists was gathered for the Reality study.

## Methods and analysis

### Study design

The Reality study is an observational and retrospective analysis, based on electronic medical registries (EMR) from the BIG-PAC^®^ database (18). This database includes the EMR of 1.8 million people from 7 health areas (including public primary care centers and hospitals) of 7 Autonomous Communities in Spain. EMR are anonymized in the source centers, according to the Organic Law 3/2018, 5th December of Protection of personal data and guarantee of digital rights (19). The BIG-PAC^®^ database is representative of the Spanish population (20).

### Objectives

The primary objective is to describe the demographic and clinical characteristics, comorbidities, and concomitant medications of patients with ASCVD and hypercholesterolemia and of patients with FH. The secondary objectives are (1) to describe the lipid-lowering treatment to reduce cholesterol in patients with ASCVD and hypercholesterolemia and patients with FH, (2) to describe the treatment modifications, the reasons for change, and the medication persistence during the follow-up period, along with the characteristics of non-adherent patients (medication possession ratio [MPR] <80%), (3) to evaluate the LDL levels during the follow-up period, according to the intensity of the lipid-lowering treatment, (4) to estimate the prevalence and the incidence rates of ASCVD and FH, (5) to estimate the use of healthcare resources and costs during the follow-up period and (6) to estimate the utilization of healthcare resources in primary care centers and hospitals during the follow-up period. The exploratory objectives include estimating the percentage of patients who are intolerant to statins and to assess the use of resources and costs associated with ASCVD and FH, according to the intensity of the lipid-lowering treatment and the LDL levels.

### Study population

The study includes patients who had a new or recurrent episode of ASCVD during the recruitment period (from 01/01/2017 to 31/12/2018). The index date will be defined as the date of the ASCVD event, and the follow-up period is 24 months after the index date. If a patient with recurrent events suffers an ASCVD event during the recruitment period, the date of the first event during that period will be considered as the first event. The diagnoses of ASCVD and hypercholesterolemia will be obtained from the International Classification of Diseases, 9th edition, Clinical Modification (ICD-9-CM) (21) coding system: (1) stable/unstable angina (codes: 411, 413), (2) acute myocardial infarction (AMI) (codes: 410, 412), (3) ischemic stroke (codes: 433, 434, 436), (4) transient ischemic attack (TIA, code: 435), (5) peripheral arterial disease (PAD, codes: 440 - 441, 444) and (6) FH (code: 272.0). Patients will be classified as ASCVD (5 groups) or FH, according to their first diagnosis in the database.

The diagnostic criteria are based on the patients' symptoms, in agreement with medical practice. In addition, diagnostic tests are used to detect structural or functional abnormalities in the heart, brain, kidney, or blood vessels.

The inclusion criteria are (a) age ≥18 years; (b) diagnosis of ASCVD or FH during the recruitment period; (c) being active in the database (defined as having ≥2 records at least 6 months before the index date; (d) having ≥2 records during the follow-up period and (e) patients with ASCVD should have at least a record of LDL ≥70 mg/dL at any time before or 3 months after the index date, diagnosis of hypercholesterolemia or lipid-lowering treatments. Patients who are not correctly identified in the database (e.g., birth date or sex have not been recorded) are not included in the study.

### Subgroups

Regardless of their **first** diagnosis in the database, patients with ASCVD and hypercholesterolemia will be divided into (1) those who received statins, (2) those who were not on treatment with statins, and (3) those who were on other treatments to reduce cholesterol. The ability of the lipid-lowering treatment to reduce cholesterol (extreme, very high, high and moderate) will be also considered in this group of patients, according to the Consensus document of the Spanish Society of Cardiology (22) (Table 1). Patients will be further classified considering their LDL levels at the time they received a ASCVD diagnosis after the earliest statin prescription. The cutoff values will be 55 mg/dL, 70 mg/dL and 100 mg/dL.

**Table 1:** Classification of lipid-lowering drugs according to their ability to reduce cholesterol.

**Lowering ability**	**Treatments**
Extreme	Evolocumab 140 mg
	Alirocumab 75 mg
	Alirocumab 150 mg
Very high	*Potent statin + ezetimibe*
	Atorvastatin 40–80 mg + ezetimibe 10 mg
	Rosuvastatin 10–40 mg + ezetimibe 10 mg
High	*High-potency statin*
	Atorvastatin 40–80 mg
	Rosuvastatin 20–40 mg
	Medium-potency statin + ezetimibe
	Simvastatin 20-40 mg + ezetimibe 10 mg
	Pravastatin 40 mg + ezetimibe 10 mg
	Lovastatin 40 mg + ezetimibe 10 mg
	Fluvastatin 80 mg + ezetimibe 10 mg
	Pitavastatin 2–4 mg + ezetimibe 10 mg
	Atorvastatin 10–20 mg + ezetimibe 10 mg
	Rosuvastatin 5 mg + ezetimibe 10 mg
Moderate	*Medium-potency statin*
	Atorvastatin 10–20 mg
	Rosuvastatin 5–10 mg
	Simvastatin 20–40 mg
	Pravastatin 40 mg
	Lovastatin 40 mg
	Pitavastatin 2–4 mg
	Fluvastatin XL 80 mg
	*Low-potency statin + ezetimibe*
	Simvastatin 10 mg + ezetimibe 10 mg
	Pravastatin 20 mg + ezetimibe 10 mg
	Lovastatin 20 mg + ezetimibe 10 mg
	Fluvastatin 40 mg + ezetimibe 10 mg
	Pitavastatin 1 mg + ezetimibe 10 mg

Source: Escobar et al. (22).

Patients with FH will be classified in (1) patients who were treated with statins, (2) patients who were not on treatment with statins and (3) patients who received other treatments to reduce cholesterol. The ability of the treatment with statins to reduce cholesterol will be also analyzed. Patients with FH will be classified according to their LDL levels at the time they received a FH diagnosis after the earliest statin prescription. The cutoff points of 55 mg/dL, 70 mg/dL and 100 mg/dL will be considered. Patients with FH will be further divided in homozygous (HoFH) and heterozygous (HeFH), and per age range.

### Data

#### Demographic characteristics and comorbidities

At the index date, the following variables will be considered: age (continuous and in ranges: 18–24; 25–34; 35–44; 45–54; 55–64; 65–75; and ≥75 years), and sex.

The comorbidities of the study population will be recorded from the medical records, during the 6 months before the index date, using the ICD-9-CM coding system. The study includes the collection of cardiovascular comorbidities (arterial hypertension, atrial fibrillation [persistent, paroxysmal, and permanent/chronic]), valvulopathies (stenosis or insufficiency [aortic or mitral]), carotid stenosis, heart failure, and angina (stable/unstable) (codes: 411, 413), AMI (codes: 410, 412), atherosclerotic aortic disease, ischemic stroke (codes: 433, 434, 436), TIA (code: 435), PAD (intermittent claudication, ischemia arterial) (codes: 440–441, 444) and atherosclerotic disorders not included in the index diagnosis. Other comorbidities will be considered, such as diabetes mellitus, obesity, chronic alcoholism, hypercholesterolemia, hypertriglyceridemia, mixed dyslipidemia, chronic renal disease (stages II, IIIa, IIIb, IV and V), hepatic disease (cirrhosis, encephalopathy, cholangitis sclerosing, liver cancer), fatty liver, chronic obstructive pulmonary disease (COPD), obstructive sleep apnea syndrome (OSAS), dementia/cognitive impairment (including Alzheimer's disease), depressive disorder, malignancies, anemia, rheumatoid arthritis, and statin intolerance. In addition, the Charlson comorbidity index (23) will be used as a summary variable of the severity status of patients (Table 2). The number of patients who smoke and the corresponding percentages in each cohort will be estimated.

**Table 2:** Diagnoses included for the calculation of the Charlson comorbidity index, and their corresponding ICD-09-CM codes.

**ICD-09-CM codes**	**Condition**	**Score**
410 – 410.9, 412	Myocardial infarction	1
428 – 428.9	Congestive heart failure	1
433.9, 441 – 441.9, 785.4, V43.4	Peripheral vascular disease	1
430 – 438	Cerebrovascular disease	1
290 – 290.9	Dementia	1
490 – 496, 500 – 505, 506.4	Chronic pulmonary disease	1
710.0, 710.1, 710.4, 714.0 – 714.2, 714.81, 725	Rheumatologic disease	1
531 – 534.9	Peptic ulcer disease	1
571.2, 571.5, 571.6, 571.4 – 571.49	Mild liver disease	1
250 – 250.3, 250.7	Diabetes	1
250.4 – 250.6	Diabetes with chronic complications	2
344.1, 342 – 342.9	Hemiplegia or paraplegia	2
582 – 582.9, 583 – 583.7, 585, 586, 588 – 588.9	Kidney disease	2
140 – 172.9, 174 – 195.8, 200 – 208.9	Any malignancy, including leukemia and lymphoma	2
572.2 – 572.8	Moderate or severe liver disease	3
196 – 199.1	Metastatic solid tumor	6
042 – 044.9	AIDS	6

ICD-9-CM, International Classification of Diseases, 9th edition, Clinical Modification.

Source: Charlson et al. (23) and Deyo et al. (24).

#### Prevalence and incidence rates

Prevalence rates of ASCVD and FH will be estimated as the overall number of cases diagnosed with ASCVD or FH in the assisted population (≥18 years), during two consecutive years (2017 and 2018). Incidence rates will be calculated as the number of new cases diagnosed per 1,000 patients/year during those years. Due to the similarities of our database with the Spanish population pyramid, in terms of age and sex (20), these results will not be standardized. Prevalence and incidence rates will be provided per type of ASCVD or FH, age range (18–44 years, 45–64 years and ≥65 years) and sex. In addition, prevalence rates will be estimated by year.

#### Biochemical and anthropometric parameters

Other parameters of the study population will be collected, such as body mass index (BMI), systolic arterial pressure (SAP), diastolic arterial pressure (DAP), cholesterol, very low-density lipoproteins (VLDL), LDL, high-density lipoproteins (HDL), triglycerides, and glycoside hemoglobin (HbA1c) in diabetic patients. The levels of glutamic oxaloacetic transaminase (GOT), glutamic-pyruvic transaminase (GPT), alkaline phosphatase, and lipoprotein (a) will also be considered. These values will be analyzed at the index date, at 12 months and 24 months of follow-up.

Patients will be classified according to their LDL values at the index date into <55, 55–69, 70–99, and >100 mg/dL. In addition, the absolute (mg/dL) and relative variation (%) of the LDL levels from the index date to the last record available will be estimated. Patients with LDL levels below 55 mg/dL at the end of the study will be estimated (3). Other cutoff values will be 70 and 100 mg/dL.

#### Treatment: Procedures

The study considers the therapeutic procedures that patients had received in the 6 months before the index date. These procedures are dialysis, lipoprotein apheresis, coronary artery bypass, percutaneous coronary intervention, carotid revascularization (angioplasty without stent/endarterectomy) and thrombectomy.

#### Treatment: Lipid-lowering medication

The use of lipid-lowering medication will be obtained from the dispensation records, using the Anatomical Therapeutic Chemical Classification System (ATC) codes (25). Drugs were prescribed according to medical practice. Drug groups were fibrates (C10AB), bile acid sequestrants (C10AC) nicotinic acid (C10AD), and other lipid-modifying agents like PCSK9 inhibitors (evolocumab [C10AX13] and alirocumab [C10AX14]) (Supplementary Table S1). The use of lipid-lowering medication will be estimated as the percentage of patients who received the treatments dispensed in the 6 months before the index date, and during the follow-up period, at 12 and 24 months from the index date.

The duration of the lipid-lowering treatment will be assessed from the date when patients started the treatment to the discontinuation date, which will be defined as the date of the end of the follow-up period (24 months), the patients' death, or change to another lipid-lowering treatment or the withdrawal of treatment (≥60 days without renewing the initial medication dispensed at the community pharmacy and/or ≥2 prescriptions). Treatment modifications (e.g., doses or the addition of lipid-lowering drugs) will be described, although they are not considered reasons of discontinuation. In case of patients who received a therapy combination, it will be considered that the medication changed if the prescription of any of the molecules was modified. The persistence to the treatment will be calculated at 12 and 24 months. The adherence to the treatment will be estimated as MPR, according to the Professional Society for Health Economics and Outcomes Research (26). It will be calculated as the number of days covered by the dispensed medication divided by the number of treatment days. According to the MPR values at the end of the study, patients will be classified as adherent (MPR ≥80%) or non-adherent (MPR <80%) to the treatment. In addition, the specialty of physicians who prescribed the lipid-lowering treatment for the first time and those who modified the initial treatment will also be reported.

The use of concomitant medications will be obtained in the 6 months before the index date. Concomitant medications include drugs used in diabetes (A10), antithrombotic agents (B01), antihypertensives (C02), agents acting on the renin-angiotensin system (C09), anti-inflammatory and antirheumatic products (M01) and drugs for peptic ulcer and gastroesophageal reflux disease (GERD) (A02B).

#### Incidence of ASCVD events and mortality

To estimate the clinical effectiveness, the ASCVD events will be assessed during the follow-up period and will be divided into ischemic cardiopathy (angina [CIE-09-CM codes: 411, 413] and AMI [CIE-09-CM codes: 410, 412]), ischemic stroke [CIE-09-CM codes: 433, 434, 436], TIA [CIE-09-CM codes: 435], and PAD (CIE-09-CM codes: 440–441 and 444). All-cause and CV-cause mortality rates will be also estimated.

#### Use of healthcare resources and costs

The perspective of the Spanish National Health System and society will be considered to estimate healthcare and non-healthcare (indirect) costs during the follow-up period of the study. Healthcare costs are those related to healthcare resources (primary care visits, emergency visits, hospitalizations [annualized rate of hospitalizations and length of the stay], specialized care visits [Cardiology, Internal medicine, Endocrinology, Vascular Surgeon, Neurology, Rehabilitation and Geriatrics departments], complementary tests (laboratory tests, conventional radiology, computed tomography [CT], nuclear magnetic resonance [NMR], catheterization, angioplasty, endarterectomy and thrombectomy) and medication. Healthcare costs will be calculated considering the frequency of use during the follow-up and their unit cost. Unit costs were estimated as the mean of the official tariffs from the Spanish Regions [when necessary, official tariffs were updated to 2021, according to the gross domestic product [GDP] (27)] (Table 3 and Supplementary Table S2). Drug costs will be quantified using the retail price per pack at the time of dispensing from the community pharmacy (29).

**Table 3:** Unit costs (year 2021).

**Resources**	Unit costs (€)^*^
Healthcare resources	
Medical visits	
Primary care	49.17
Specialized care^**^	101.9
Emergency department	258.92
Hospitalization (per day)	631.54
Day hospital sessions^***^	4,483.17
Complementary tests	
Laboratory tests	13.49
Conventional radiology	21.47
Computed tomography	240.53
Nuclear magnetic resonance	315.19
Other diagnostic/therapeutic tests^****^	154.00
Pharmaceutical prescription	Retail price
Labor productivity (indirect costs)	
Cost per day of sick leave	101.21

* Unit costs related to the management of ASCVD were identified.

** It considers Cardiology, Internal medicine, Endocrinology, Vascular, Neurology, Rehabilitation and Geriatrics departments.

*** It includes catheterization, angioplasty, endarterectomy/thrombectomy.

**** It includes echocardiogram, cardiac exercise stress tests and Holter monitor tests.

ASCVD, arteriosclerotic cardiovascular disease.

Source: Mean of the official tariffs from the Spanish Regions and INE (28).

Non-healthcare costs include those associated with lost productivity (days of sick leave due to temporary or permanent disability in the working population [<65 years]) and premature deaths. Productivity losses will be calculated taking into account the number of sick leave days/permanent disability days, the percentage of patients who were on sick leave/permanent disability and the mean salary of the Spanish population, according to the National Institute of Statistics (28) (Table 3). To estimate the non-healthcare costs associated with premature deaths, the total number of lost days per patient (difference between the death age and 65 years, which is the retirement age in Spain) and the average salary of the study population (women: €21,251; men: €27,334) will be considered (28).

The use of healthcare resources and costs will be estimated at 12 and 24 months and costs will be expressed in Euros for the year 2021. Direct non-healthcare costs will not be considered. Healthcare costs and indirect costs will be assessed in patients with ASCVD (5 groups) and FH.

#### Statistical analysis

The statistical analysis will be exploratory and descriptive. The study is not designed to confirm or reject predefined hypotheses. Computer statements (SQL script) will be used to obtain data from the database. Data will be reviewed, using exploratory analyses, checking the distributions, and looking for possible recording or codification mistakes. Data will be validated to guarantee the quality of the results. It is estimated that, based on the inclusion/exclusion criteria, the overall study population will include more than 2,600 patients. The results will be provided for the 6 subgroups of patients previously described.

A statistical univariate analysis for the variables of interest will be carried out. Qualitative data will be described using absolute and relative frequencies (N, %), and quantitative data by using mean and standard deviation (SD) in symmetric distributions and median (interquartile ranges, Q1 – Q3) in asymmetric distributions. In addition, 95% confidence intervals (CI) will be provided to estimate the population parameters. Bivariate analyses (ANOVA and Chi2 tests) will be carried out to compare independent groups regarding statin treatment and LDL levels. The persistence/duration of treatment will be estimated by using a Kaplan-Meier survival analysis (procedure: log-rank test).

Paired statistical tests will be used to estimate the anthropometric/biochemical parameters at the beginning and the end of the study (McNemar/t-Student tests). An ANOVA (generalized lineal model) will be developed to adjust costs for sex, age, Charlson index and time since diagnosis (procedure: marginal means; Bonferroni adjustment). Sub-analyses may be carried out according to new lines of interest. A 0.05 level of statistical significance will be applied in all statistical tests. Data will be analyzed using the SPSS (v27.0) statistical package (SPSS Inc., Chicago, Illinois, USA).

## Discussion

This study aims to evaluate the treatment patterns and use of healthcare resources of patients with ASCVD and hypercholesterolemia and patients with FH in Spain. It will include patients who suffered an ASCVD event between 01/01/2017 and 31/12/2018, were attended at public primary care centers or hospitals and registered in the BIG-PAC^®^ database. The patients' characteristics, treatment, comorbidities, use of resources and costs will be analyzed during the study period.

Previous observational studies analyzed the management of dyslipidemia in patients with coronary artery disease in Spain. Recently, Alarcón et al. carried out a study in 200 patients with ischemic heart disease during a follow-up period of 5 years. They showed that just 21.7% of them met the optimal therapeutic objective, as they had stopped smoking, were on treatment with statins and aspirin and had LDL levels <70 mg/dL (30). The acute coronary syndrome (ACS) patient pathway project also concluded that the lipid management in these patients is suboptimal, since at least half of them had LDL levels >70 mg/dL and most of their treatments had not been modified during the follow-up period (31). In line with these results, Ribas et al. reported that just 45% of ACS patients reached LDL levels <70 mg/dL in the 122 days after the ACS, and that the use of Masana's recommendations improved the management and treatment planification in these patients (11). In addition, Ródenas et al. observed that only one third of patients who had suffered a previous myocardial infarction had LDL levels <70 mg/dL during a follow-up period between 2013 and 2018 (32). These analyses considered a relatively small population of patients, but it is expected that our study will include more than 2,600 patients with ASCVD. Therefore, our research will cover different ASCVD events, so that the patients' characteristics, lipid profiles, treatments, use of resources and costs may be compared.

On the other hand, Redón et al. analyzed the secondary prevention therapies administered during the first year after the hospital admission (due to stroke, myocardial infarction, or coronary revascularization) between 01/01/2011 and 31/12/2013, of 92,432 patients in the Valencian Community (33). Nevertheless, our study will provide more updated information about the management of these patients, using a database representative of the Spanish population (20).

Regarding the heterozygous FH, the SAFEHEART study recorded 2,648 patients in Spain. This recent study estimated the incidence of CV events, the use of lipid-lowering treatments and the achievement of treatment goals among other objectives (34). Due to the design of the Reality study, it will consider a smaller population of patients with FH, but our results will add more information on the use of healthcare resources, and direct healthcare and indirect costs associated with the management of these patients.

The main contributions of this study are the use of real-world data, and the consideration of a wide population of patients with ASCVD. It will provide useful information about the prevalence and incidence rates of ASCVD and FH, the characteristics of these patients and the effectiveness of the treatments currently available. To our knowledge, this will be the first study that estimates the indirect costs associated with ASCVD and FH in Spain. However, our study is not without limitations. First, BIG-PAC^®^ is an administrative database and may have missing information about the study population, particularly if patients have been treated in private centers or public centers that are outside of its area of influence. Second, this study might have the usual limitations of retrospective studies, such as under-recording of diseases (particularly regarding the diagnosis of unstable angina and TIA), the possible inaccuracy of codification, or possible variations in medical practice among professionals. As it was revealed in previous studies, the ICD-9-CM coding system may be poor to identify incident cases of stroke and it should be used in combination with additional data, like physician notes or imaging studies, to confirm the diagnosis of valvular diseases and vascular disorders (35, 36). However, this codification system is widely used in most Spanish hospitals. Third, it should be noted that the BIG-PAC^®^ database may lack variables that could influence our results, such as the socioeconomic level of patients. Finally, since the study lasted until 31/12/2020, our results may be affected by the COVID-19 pandemic. This effect will be estimated in future sub analyses.

In conclusion, this study aims to analyze the treatment patterns and use of healthcare resources ASCVD and FH in Spain. The prevalence of these disorders will also be provided. Due to the high morbidity and mortality associated with these diseases, it is expected that our study will provide useful information for healthcare systems and decision makers to improve the management of these disabling diseases.

## Ethics statement

The studies involving human participants were reviewed and approved by Ethics Committee of the Hospital of Terrassa (Barcelona). Written informed consent for participation was not required for this study in accordance with the national legislation and the institutional requirements.

## Author contributions

The study was conceived by AS-M, AS, NM, and JP. Participated and contributed to the design of the study by VB, MC, RC, JG, IE, JMG, IM, JM, VP-C, AS-M, NM, and JP. Data collection and statistical analysis was made by AS-M and AS. The first draft of the manuscript was written by IP. All authors reviewed critically and approved the final version of the submitted manuscript.

## Funding

This study was funded by Novartis.

## Conflict of interest

This study is a Research collaboration between Atrys Health SA and Novartis Pharmaceuticals. Author VB has received consultancy and lecturing/speaker bureau fees from Almirall, Amgen, Daiichi-Sankyo, Novartis, MSD/Organon, Sanofi, Servier and Mylan/Viatris. Author MC has received consultancy fees for consulting services and as a speaker from Ferrer, Amgen, Daiichi-Sankyo, BMS-Pfizer, Böehringer-Ingelheim, Astra Zeneca, Alexion, Novartis and Lundbeck. Author RC has received lecture fees from Amgen, Daiichi-Sankyo, Ferrer, Mylan, MSD, Organon, Sanofi, Servier and Novartis. She has participated in consultancies for Novartis, Amgen, Sanofi and Servier. She has also received collaborations to scientific or research projects from Amgen, Daiichi-Sankyo, Ferrer, Organon, Servier and Novartis. Author JG declares to have received honoraria for oral presentations and advisory boards, and research grants from Almirall, Novartis, Amgen, Sanofi, and Daiichi. Author IE declares to have received fees for consulting services from Novartis, Servier, and Boehringer-Ingelheim, and to have received fees for services as a speaker from Novartis, Vifor, and Boehringer-Ingelheim. Author JMG declares to have received fees for consulting services from Novartis, Daiichi-Sankyo, Almirall, Sanofi, and Böehringer-Ingelheim, and to have received fees for services as a speaker from Novartis, Daiichi-Sankyo, Almirall, Sanofi, Amgen, Organon, Viatris, Rovi and Menarini. Author JM has received fees for consulting services from Pfizer, Amaryn, Novartis, Daiichi-Sankyo, Servier, Sanofi, and Amgen, and has received fees for services as a speaker from Novartis, Daiichi-Sankyo, Servier, Sanofi, Amgen, Viatris, and Alter. Author VP-C has received fees for consulting and speaker bureau from Almirall, Daiichi-Sankyo, Novartis, Sanofi, Almirall, Rovi, Menarini, Organon. The remaining authors declare that the research was conducted in the absence of any commercial or financial relationships that could be construed as a potential conflict of interest.

## Publisher's note

All claims expressed in this article are solely those of the authors and do not necessarily represent those of their affiliated organizations, or those of the publisher, the editors and the reviewers. Any product that may be evaluated in this article, or claim that may be made by its manufacturer, is not guaranteed or endorsed by the publisher.
