# Supplementary material for: Treatment patterns and use of healthcare resources of patients with atherosclerotic cardiovascular disease and hypercholesterolemia and patients with familial hypercholesterolemia in Spain: Protocol of the Reality study
Source: Front Cardiovasc Med. 2022 Aug 4;9:966049. doi: 10.3389/fcvm.2022.966049 (PMC9386132; doi:10.3389/fcvm.2022.966049)
Supplement: Supplementary file 1 [file Table_1.DOCX]

Supplementary Material

# Supplementary Tables

Table S1. Lipid-modifying agents.

| C10A Lipid modifying agents | |
| --- | --- |
| C10AA HMG CoA reductase inhibitors | C10AA01 Simvastatin  C10AA02 Lovastatin  C10AA03 Pravastatin  C10AA04 Fluvastatin  C10AA05 Atorvastatin  C10AA06 Cerivastatin  C10AA07 Rosuvastatin  C10AA08 Pitavastatin |
| C10AB Fibrates​ | C10AB01 Clofibrate  C10AB02 Bezafibrate  C10AB03 Aluminium clofibrate  C10AB04 Gemfibrozil  C10AB05 Fenofibrate  C10AB06 Simfibrate  C10AB07 Ronifibrate  C10AB08 Ciprofibrate  C10AB09 Etofibrate  C10AB10 Clofibride |
| C10AC Bile acid sequestrants | C10AC01 Colestyramine  C10AC02 Colestipol  C10AC03 Colextran  C10AC04 Colesevelam |
| C10AX Other lipid modifying agents | C10AX01 Dextrotiroxina.  C10AX02 Probucol.  C10AX03 Tiadenol.  C10AX04 Benfluorex  C10AX05 Meglutol  C10AX06 Omega-3-triglycerides incl. other esters and acids  C10AX07 Magnesium pyridoxal 5-phosphate glutamate  C10AX08 Policosanol.  C10AX09 Ezetimiba  C10AX10 Alipogene tiparvovec  C10AX11 Mipomersen  C10AX12 Lomitapide  C10AX13 Evolocumab  C10AX14 Alirocumab  C10AX15 Bempedoic acid |
| C10B Lipid modifying agents in combination | |
| C10BA HMG CoA reductase inhibitors in combination with other lipid modifying agents | C10BA01 Lovastatin and nicotinic acid  C10BA02 Simvastatin and ezetimibe  C10BA03 Pravastatin and fenofibrate  C10BA04 Simvastatin and fenofibrate  C10BA05 Atorvastatin and ezetimibe  C10BA06 Rosuvastatin and ezetimibe |
| C10BX HMG CoA reductase inhibitors, other combinations | C10BX01 Simvastatin and acetylsalicylic acid  C10BX02 Pravastatin and acetylsalicylic acid  C10BX03 Atorvastatin and amlodipine  C10BX04 Simvastatin, acetylsalicylic acid and ramipril  C10BX05 Rosuvastatin and acetylsalisylic acid  C10BX06 Atorvastatin, acetylsalicylic acid and ramipril  C10BX07 Rosuvastatin, amlodipine and lisinopril  C10BX08 Atorvastatin and acetylsalicylic acid  C10BX09 Rosuvastatin and amlodipine  C10BX10 Rosuvastatin and valsartan  C10BX11 Atorvastatin, amlodipine and perindopril  C10BX12 Atorvastatin, acetylsalicylic and perindopril  C10BX13 Rosuvastatin, perindopril and indapamide  C10BX14 Rosuvastatin, amlodipine and perindopril  C10BX15 Atorvastatin and perindopril |

*Source: The Anatomical Therapeutic Chemical Classification System*[24]

Table S2. Sources of the official tariffs from the Spanish Regions.

| **Autonomous Community** | **Source** |
| --- | --- |
| Andalusia | Boletín Oficial Junta Andalucía num. 210 |
| Aragon | Boletin Oficial de Aragón num. 156 |
| Asturias | Boletín Oficial Principado de Asturias num. 77 |
| Balearic Islands | Boletin Oficial Islas Baleares num. 2 |
| Canary Islands | Boletín Oficial de Canarias num. 67 |
| Cantabria | Boletín Oficial de Cantabria num. 248 |
| Castilla La Mancha | Diario Oficial Castilla La Mancha num. 226 |
| Castile and León | Boletin Oficial de Castilla y León num. 249 |
| Catalonia | Diario Oficial Generalidad Cataluña num. 6387 |
| Extremadura | Boletin Oficial de Extremadura num. 35 |
| Galicia | Diario Oficial de Galicia num. 96 |
| La Rioja | Boletin Oficial de La Rioja num. 156 |
| Community of Madrid | Boletin Oficial Comunidad Madrid num. 198 |
| Region of Murcia | Boletín Oficial Región de Murcia num. 48 |
| Navarre | Boletín de Navarra num. 14 |
| Basque Country | Tarifas para facturación de servicios sanitarios y docentes de Osakidetza para el año 2020 |
| Valencian Community | DOGV num 8202 Ley de Tasas Comunidad Valenciana - Tasas Sanidad (pag 49735) |
